# Magnesium prophylaxis of new-onset atrial fibrillation: A systematic review and meta-analysis

**DOI:** 10.1371/journal.pone.0292974

**Published:** 2023-10-26

**Authors:** Jeffrey Curran, Amanda Ross-White, Stephanie Sibley

**Affiliations:** 1 Department of Critical Care Medicine, Queen’s University, Kingston, Canada; 2 Bracken Health Sciences Library, Queen’s University, Kingston, Canada; University of Arizona Medical Center - University Campus: Banner University Medical Center Tucson, UNITED STATES

## Abstract

**Purpose:**

Atrial fibrillation (AF) is the most common cardiac arrhythmia in intensive care units (ICU) and is associated with increased morbidity and mortality. Magnesium prophylaxis has been shown to reduce incidence of AF in cardiac surgery patients, however, evidence outside this population is limited. The objective of this study is to summarize studies examining magnesium versus placebo in the prevention of NOAF outside the setting of cardiac surgery.

**Source:**

We performed a comprehensive search of MEDLINE, EMBASE, and Cochrane Library (CENTRAL) from inception until January 3^rd^, 2023. We included all interventional research studies that compared magnesium to placebo and excluded case reports and post cardiac surgery patients. We conducted meta-analysis using the inverse variance method with random effects modelling.

**Principal findings:**

Of the 1493 studies imported for screening, 87 full texts were assessed for eligibility and six citations, representing five randomized controlled trials (n = 4713), were included in the review, with four studies (n = 4654) included in the pooled analysis. Administration of magnesium did not significantly reduce the incidence of NOAF compared to placebo (OR 0.72, [95% CI 0.48 to 1.09]).

**Conclusion:**

Use of magnesium did not reduce the incidence of NOAF, however these studies represent diverse groups and are hindered by significant bias. Further studies are necessary to determine if there is benefit to magnesium prophylaxis for NOAF in non-cardiac surgery patients.

## Introduction

New-onset atrial fibrillation (NOAF) is the most common arrhythmia in critically ill patients with a reported incidence of 20% [1, 2]. It is commonly described in patients with sepsis [3–6] but is seen in a variety of illnesses such as acute respiratory distress syndrome [7], non-cardiac thoracic surgery [8–10], and trauma [11, 12]. NOAF may result in hemodynamic instability, and is often difficult to resolve while critical illness is ongoing [13]. Development of NOAF is associated with elevated risk of in-hospital ischemic stroke [14, 15], and mortality [16]. Eighteen percent of NOAF patients who survive to ICU discharge have ongoing atrial fibrillation (AF) [17]. Of the patients who develop NOAF but convert to normal sinus rhythm (NSR) before ICU discharge, a significant number will develop recurrent AF and have elevated risk of ischemic stroke, heart failure, and death within five years [18].

Prophylaxis of NOAF in the ICU has potential to improve patient outcomes with decreased hemodynamic instability, exposure to medications with potential side effects, uncomfortable electrical cardioversion attempts, decreased risk of stroke, heart failure and mortality, and cost-savings [19]. Few prophylactic interventions for NOAF have been studied in a critical care population, with two small observational studies in ICU patients suggesting that hydrocortisone use was associated with a decreased risk of developing NOAF in the acute phase of critical illness [20, 21], and one study comparing liberal versus restrictive magnesium supplementation for prevention of atrial fibrillation [22]. This is in contrast to cardiac surgery populations, where pharmacologic and surgical prophylaxis against NOAF has been well studied and has been successful in reducing the incidence of AF, as well as postoperative morbidity and mortality [23]. One preventative approach in cardiac surgery patients is the use of magnesium sulphate, and several meta-analyses have shown reduced rates of AF with this intervention [24–31].

Critically ill patients have many risk factors for the development of NOAF including a high prevalence of hypomagnesemia [32, 33], heightened inflammatory state [34, 35], and frequent use of vasoactive medications [36, 37]. Despite the potential benefit of magnesium prophylaxis in critically ill patients due to its anti-arrhythmogenic [38] and anti-inflammatory [39, 40] properties, magnesium prophylaxis of NOAF in populations outside of cardiac surgery has not been adequately studied. Due to the lack of evidence to guide practice on the use of magnesium in a general critical care population, we performed a systematic review and meta-analysis to determine if there was a benefit for the use of magnesium therapy for the prophylaxis of NOAF in any patients outside the cardiac surgery population, including subgroups that may be found in a medical/surgical ICU to inform practice in this population.

## Methods

This systematic review is reported in accordance with the PRISMA guidelines [41] and was registered with PROSPERO (ID: CRD42021273482; submitted: August 13, 2021; registered: September 13, 2021). PRISMA checklist can be found in the supporting information (S1 Checklist).

### Search strategy and selection process

We searched the following electronic databases separately on the Ovid platform: MEDLINE, EMBASE, and Cochrane Library (CENTRAL) from inception until January 3, 2023. We limited the search to non-animal studies, non-child studies and English language only. We checked the reference lists of review articles to identify other relevant studies. The search strategy was developed in collaboration with a Health Information Specialist using Medical Subject Heading (MeSH) terms and keywords related to magnesium supplementation and atrial fibrillation. The full search strategy is available in the supporting information (S1 Appendix).

We included all research studies that compared magnesium to placebo for the prevention of atrial fibrillation, excluding case reports. Studies of cardiac surgery patients were excluded. We included the following outcomes of interest: incidence of new onset atrial fibrillation and rate of premature atrial contractions.

We used Covidence online platform (www.covidence.org) for screening. We combined search results from all sources and removed duplicates using the Covidence algorithm. Screening was performed in duplicate by two reviewers (J.D.C. and S.S.) using a two-stage screening approach: title and abstract screening then full-text review. Any potentially eligible citation identified by either reviewer was advanced to stage two. The same reviewers evaluated full texts with disagreements resolved through consensus.

### Data collection, risk of bias, and certainty assessment

Data was extracted using Covidence online platform that included author, date, population characteristics, interventions, and outcomes of interest. Risk of bias (ROB) for included studies was assessed using the Cochrane ROB Tool. Two reviewers (J.D.C. and S.S.) extracted data and assessed ROB independently and in parallel with disagreements resolved by discussion and consensus.

We assessed overall certainty of evidence using Grading of Recommendations, Assessment, Development and Evaluations (GRADE) methodology [42].

### Data analysis

Meta-analysis was conducted using the inverse variance method with random effects modelling. All analyses were performed using the RevMan 5.4 software (www.revman.cochrane.org). We present effect measures as odds ratios (OR) with associated 95% confidence intervals (CI). We assessed heterogeneity using the chi-square test, the I^2^ statistic. The limited number of studies that met inclusion criteria precluded pre-planned subgroup analysis on dose and route of magnesium administration and critically ill patients.

## Results

### Search strategy and study characteristics

**Fig 1:**
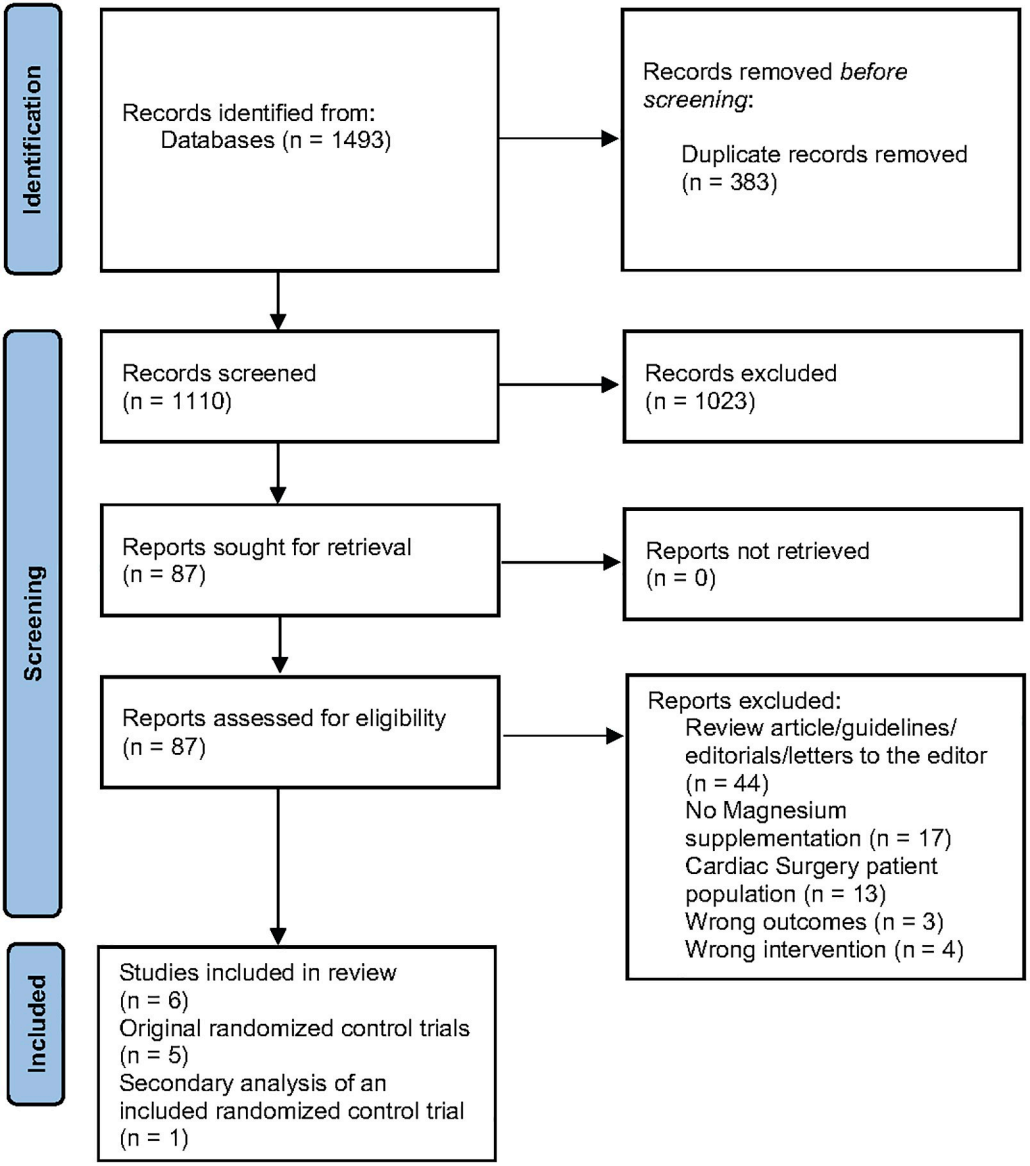
PRISMA flow chart.

A total of 1493 studies were imported for screening with 1110 studies screened after duplicates removed. Eighty-seven full-text studies were assessed for eligibility, with six citations representing five randomized controlled trials (RCTs) (n = 4713) included in the review as shown in Fig 1 [43–48]. Four studies (n = 4654) were included in the pooled analysis. The dose and form of magnesium administered varied between studies, as well as the duration of cardiac monitoring (Table 1).

**Table 1:** Characteristics of included studies.

Author/Year	Country	Population	Number of participants		Mean age		N Male (%)		Intervention	Additional Interventions	Duration of cardiac monitoring/ICU admission	Methods of monitoring	Primary Outcome
			Mg	Placebo	Mg	Placebo	Mg	Placebo					
Roffe 1994	United Kingdom	Acute myocardial infarction	1159	1157	61.4	62.2	855 (74%)	846 (73%)	8 mmol magnesium sulphate over five minutes + 65 mmol over 24 hours vs equal volumes of saline		While admitted to the coronary care unit (48 hours on average)	Continuous ECG monitoring or 24-hour Holter monitor	Frequency of cardiac arrhythmias
Terzi 1996	Italy	Post-thoracic surgery	95	105	65	63	Not reported	Not reported	2 g (16mEq) magnesium sulphate bolus infusion at thoracotomy + 2 g after 6 hours vs no treatment	Control patients received Digoxin (0.5mg) on day of surgery if age > 70 years or undergoing pneumonectomy or intrapericardial procedure	Not reported	Continuous ECG monitoring for at least 24 hours and usually 48 hours after pneumonectomy, 12- lead electrocardiogram on POD 1 and 2, thereafter on clinical suspicion of dysrhythmia	Development of atrial tachy-arrhythmias
Khalil 2013	Egypt	Patients undergoing lobectomy	219	219 (Historical controls)	64.2	63.7	154 (70%)	149 (68%)	80 mg/kg magnesium sulphate bolus preoperatively followed by 8 mg/kg/hr x 48 hours vs no treatment	All hypertensive patients were on preoperative selective beta-blockers and/or calcium channel blocker treatment	ICU admission post-operatively	Continuous ECG monitoring while in ICU, daily 12- lead electrocardiogram; additional 12-lead electrocardiogram with palpitations or irregular pulse	Development of atrial fibrillation
Saver 2015 (Bechler 2020)	United States	Acute stroke	857	843	69	69	499 (58%)	476 (56.5)	4 g magnesium sulphate bolus pre-hospital followed by infusion of 16 g over 24 hours		Not reported	Heart rate recorded every 4 hours for the first 24 hours, then every 8 hours for the second 24 hours	Disability at 3 months by Rankin Score Cardiac adverse events
Lutsey 2018	United States	Healthy volunteers aged 55 or older	29	30	61.3	61.6	16 (27.1)	4 (13.8)	400 mg of magnesium oxide or placebo orally		Zio Patch for 2- weeks pre-randomization, then for 2-weeks 10-weeks after completing the study drug	Zio Patch (iRhythm Technologies Inc, San Francisco, USA)	Feasibility metrics; Premature atrial contraction burden

Risk of bias (ROB) was judged to be high in three studies stemming from a lack of blinding and use of historical controls (Khalil 2013, Terzi 1996) and selective reporting (Roffe 1994). ROB of included studies is shown in Table 2.

**Table 2:** Risk of bias summary using Cochrane risk of bias tool.

First author, year	Sequence Generation	Allocation concealment	Blinding of outcome assessors	Blinding of participants and personnel	Incomplete outcome data	Selective outcome reporting	Other sources of bias
Roffe, 1994	?	+	+	+	+	-	-
Terzi, 1996	?	?	-	-	+	+	+
Khalil, 2013	+	+	-	-	+	+	-
Lutsey, 2018	+	+	+	+	+	+	+
Saver, 2015	+	+	+	+	+	+	+

Symbols represent high (-), unclear (?), or low (+) risk of bias.

### Pooled analysis

**Fig 2:**
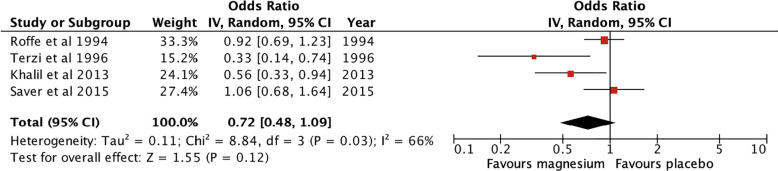
Forest plot of magnesium vs placebo in the prevention of atrial fibrillation.

Administration of magnesium compared to placebo did not have a statistically significant effect on incidence of NOAF compared to placebo (OR 0.72, 95% CI 0.48 to 1.09). Forest plot of ORs shown in Fig 2. The included studies had substantial heterogeneity (I^2^ = 66%). A leave-one-out sensitivity analysis was performed and demonstrated the thoracic surgery studies contributed the greatest positive effect of magnesium supplementation, however the result remained statistically insignificant regardless of which study was removed (S1 Table).

Overall certainty of evidence was judged to be very low stemming from concerns related to risk of bias and sparsity of trials that examined outcomes of interest and indirectness to our population of interest. (S2 Table).

One study of oral magnesium in healthy volunteers [48] was not included in the pooled analysis as they examined premature atrial contractions which could not be directly compared to incidence of new atrial fibrillation. In this study there was no difference in change in log premature atrial contraction burden among participants.

## Discussion

This systematic review and meta-analysis found that administration of magnesium did not result in a statistically significant reduction in the incidence of NOAF compared to placebo in non-cardiac surgery patients. The certainty of the results was judged to be very low due to high risk of bias and heterogeneity in the patient populations, interventions, and detection of NOAF in the included studies.

These findings are in contrast with several cardiac surgery meta-analyses which have demonstrated a reduction in the incidence of post-operative atrial fibrillation (AF) and decreased morbidity and mortality [24–31] with magnesium administration. Fairley *et al* reported a reduction in post-operative AF (RR 0.69, [95% CI 0.56–0.86]), with an even greater effect when the magnesium was given post-operatively and for longer than 24 hours (RR 0.51, [95% CI 0.34–0.77) [49]. However, uncertainty remains due to the significant methodological heterogeneity of studies included in these analyses. A meta-analysis performed by Cook *et al* restricted included studies to five double-blind, intention to treat studies where AF was the primary outcome. Heterogeneity was not significant, and no difference in rates of post-operative AF were seen (OR 0.94, p = 0.77) [50]. Similarly, Chaudhary *et al* did not show a difference in the overall rates of post-operative AF, however a subgroup analysis showed a significant reduction if the magnesium supplementation was given post-operatively (RR 0.76; [95% CI 0.58–0.99]) [51]. The variability in these results demonstrates the importance of dose, timing, and duration of administration of magnesium supplementation [51].

Cardiac surgery patients differ from patients in a general medical/surgical ICU as they have higher rates of post-operative AF [31] and increased risk of arrhythmia from special anatomical and electrophysiological characteristics such as development of ectopic foci in the pulmonary veins, inflammation adjacent to surgical margins, and direct trauma to the atrial and pleural surfaces [52, 53]. However, they share characteristics with critically ill patients such as increased inflammation [54] and oxidative stress [55], and this heightened inflammatory state has been proposed to be an important driver of NOAF [34, 35]. Direct cardiac effects can be noted early after exposure to inflammatory cytokines, with structural and electrical changes due to alterations in ion channels and gap-junction forming connexins [56]. Several cytokines have been implicated in this process, including tumor necrosis factor (TNF), IL-1, and IL-6, which can rapidly induce reversible atrial electrical remodeling [40]. Additionally, a recent study has demonstrated a genetic susceptibility for the development of NOAF in critical illness associated with genes that influence expression of antioxidant proteins. These variations could affect myocardial capacity to maintain homeostasis during times of oxidative stress often seen in critical illness [57].

The prevention of NOAF in critically ill patients has the potential to improve patient-centred outcomes such as stroke and mortality and reduce utilization costs associated with prolonged ICU and hospital stays [19]. Several pharmacologic and surgical interventions for the prophylaxis of NOAF have been studied in surgical settings, demonstrating effective prevention of NOAF [23, 58]. Though drugs such as beta-blockers, amiodarone and calcium channel blockers have been effective in post-operative patients for prophylaxis of atrial arrhythmias, they carry significant potential harm as prophylactic agents and have been associated with significant hypotension and increased all-cause mortality [59]. Many patients in the ICU experience circulatory shock and require vasoactive agents, limiting the use of beta-blockers and calcium channel blockers for NOAF. Anti-inflammatory medications such as hydrocortisone have been associated with a decreased risk of developing NOAF [20, 21], but may contribute to hyperglycemia, hypernatremia and fluid retention, immunosuppression, psychiatric side-effects and ICU-acquired weakness and must be administered judiciously, limiting use as prophylaxis for NOAF [60].

Magnesium supplementation is an inexpensive intervention with a favorable safety profile [49]. The anti-arrhythmogenic effects of magnesium are believed to be mediated through several mechanisms. Parenteral magnesium can correct an altered homeostasis due to Mg deficiency [61–63]. Magnesium can raise the threshold for electrical excitation in myocardial cells, slowing atrioventricular conduction, and increasing sinus node recovery times [38], particularly in the setting of use of vasoactive medications. Magnesium may reduce inflammatory markers [39], specifically IL-6, which is uniquely associated with atrial electrical remodeling [40]. It has shown promise in preventing the development of NOAF. In a network meta-analysis among post general thoracic surgery patients, magnesium therapy was effective in prevention of post-operative atrial fibrillation (OR 0.35; 0.16 to 0.74 95% CI) compared to placebo/usual care with no obvious side effects [58]. Wilson et al performed an instrument variable analysis using the supplementation preference of bedside critical care nurses, and showed liberal magnesium supplementation was associated with a 3% decreased relative risk of experiencing an AF event when compared to restrictive supplementation [22].

This study has several limitations. A small number of studies were included, and we found no studies in general critically ill patients. However, studies included in this analysis represent subsets of patients who may overlap with patients found in medical/surgical ICUs. Several of the included studies had small sample sizes and were judged to have a high risk of bias due to a lack of blinding and selective reporting. There was significant heterogeneity in outcomes, likely due to differing patient populations, dosing and timing of magnesium sulphate, and monitoring. It is important to note the two largest studies included in the meta-analysis (Saver/Bechler and Roffe) [44] were secondary analysis of the FAST-MAG and LIMIT-2 [64] trial, whose primary outcomes were not development of atrial fibrillation. These studies were unclear about the duration of cardiac monitoring and how the diagnosis of atrial fibrillation was made retrospectively, which may have resulted in missed episodes of atrial fibrillation.

## Conclusion

This systematic review and meta-analysis did not demonstrate a difference in the incidence of NOAF in non-cardiac surgery patients treated with magnesium supplementation compared to placebo. Given the heterogeneity within the included studies and high risk of bias this estimate is uncertain. There were no studies comparing magnesium to placebo in a general ICU population to guide use in that group, and a randomized control study is warranted to determine if prophylaxis with parenteral magnesium would be of benefit in that population for the prevention of NOAF.

## Supporting information

S1 Checklist PRISMA checklist. (PDF)

S1 Appendix Search strategy. (DOC)

S1 Table Leave one out analysis. (DOCX)

S2 Table GRADE evidence summary. (DOCX)

S1 Graphical abstract (PPTX)
