# Supplementary material for: Magnesium prophylaxis of new-onset atrial fibrillation: A systematic review and meta-analysis
Source: PLoS One. 2023 Oct 26;18(10):e0292974. doi: 10.1371/journal.pone.0292974 (PMC10602269; doi:10.1371/journal.pone.0292974)
Supplement: S1 Appendix — (DOC) [file pone.0292974.s002.doc]

Appendix 1 – Search Strategy

Database: EBM Reviews - Cochrane Central Register of Controlled Trials <November 2022>

Search Strategy:

--------------------------------------------------------------------------------

1 Magnesium/ (1241)

2 exp Magnesium Compounds/ (1640)

3 magnesium*.mp. (8684)

4 or/1-3 (8744)

5 Atrial Fibrillation/ (5259)

6 atrial fib*.mp. (14732)

7 or/5-6 (14732)

8 4 and 7 (168)

9 limit 8 to english language (164)

10 limit 9 to yr="2021 -Current" (14)

***************************

Database: Embase Classic+Embase <1947 to 2022 December 30>

Search Strategy:

--------------------------------------------------------------------------------

1 Magnesium/ (104548)

2 exp Magnesium Compounds/ (2869)

3 magnesium*.mp. (198477)

4 or/1-3 (198477)

5 Atrial Fibrillation/ (98278)

6 atrial fib*.mp. (191052)

7 or/5-6 (191052)

8 4 and 7 (1090)

9 limit 8 to english language (1039)

10 exp magnesium/ (104548)

11 exp atrial fibrillation/ (112026)

12 4 or 10 (198477)

13 7 or 11 (191052)

14 12 and 13 (1090)

15 limit 14 to english language (1039)

16 limit 15 to animals (10)

17 15 not 16 (1029)

18 limit 17 to animal studies (8)

19 17 not 18 (1021)

20 limit 19 to child <unspecified age> (5)

21 19 not 20 (1016)

22 limit 21 to dc=20210823-20230103 (168)

***************************

Database: Ovid MEDLINE(R) and Epub Ahead of Print, In-Process, In-Data-Review & Other Non-Indexed Citations and Daily <1946 to December 30, 2022>

Search Strategy:

--------------------------------------------------------------------------------

1 Magnesium/ (69526)

2 exp Magnesium Compounds/ (18319)

3 magnesium*.mp. (117059)

4 or/1-3 (120836)

5 Atrial Fibrillation/ (68567)

6 atrial fib*.mp. (101898)

7 or/5-6 (101898)

8 4 and 7 (384)

9 limit 8 to animals (19)

10 8 not 9 (365)

11 limit 10 to "all child (0 to 18 years)" (7)

12 10 not 11 (358)

13 limit 12 to english language (331)

14 limit 13 to dt=20210823-20230103 (21)

***************************
