# Supplementary material for: Magnesium prophylaxis of new-onset atrial fibrillation: A systematic review and meta-analysis
Source: PLoS One. 2023 Oct 26;18(10):e0292974. doi: 10.1371/journal.pone.0292974 (PMC10602269; doi:10.1371/journal.pone.0292974)
Supplement: S1 Table — (DOCX) [file pone.0292974.s003.docx]

| Omitted Study | Odds Ratio | 95% Confidence Interval |
| --- | --- | --- |
| Roffe et al | 0.62 | 0.33, 1.17 |
| Terzi et al | 0.85 | 0.62, 1.17 |
| Khalil et al | 0.78 | 0.48, 1.27 |
| Saver et al | 0.61 | 0.35, 1.06 |

Leave-one-out sensitivity analysis
