# Supplementary material for: Magnesium prophylaxis of new-onset atrial fibrillation: A systematic review and meta-analysis
Source: PLoS One. 2023 Oct 26;18(10):e0292974. doi: 10.1371/journal.pone.0292974 (PMC10602269; doi:10.1371/journal.pone.0292974)
Supplement: S2 Table — (DOCX) [file pone.0292974.s004.docx]

| **Outcome: Development of new-onset atrial fibrillation** | | | | | | | | | | | | |
| --- | --- | --- | --- | --- | --- | --- | --- | --- | --- | --- | --- | --- |
| **Certainty Assessment** | | | | | | | **No. of Patients** | | **Effect** | | **Certainty** | **Importance** |
| **Number of studies** | **Study design** | **Risk of bias** | **Inconsistency** | **Indirectness** | **Imprecision** | **Other considerations** | **Magnesium** | **Placebo** | **Relative** | **Absolute** |  |  |
| 4 | RCT | serious | serious | serious | serious | none | 2330 | 2324 | OR 0.72 (0.48-1.09) | 0 fewer NOAF events per 1000 in the exposed group compared to the control group | ⊕◯◯◯  Very low | Important |

GRADE evidence summary
