# Supplementary material for: Magnesium prophylaxis of new-onset atrial fibrillation: A systematic review and meta-analysis
Source: PLoS One. 2023 Oct 26;18(10):e0292974. doi: 10.1371/journal.pone.0292974 (PMC10602269; doi:10.1371/journal.pone.0292974)
Supplement: S1 Graphical abstract — (PPTX) [file pone.0292974.s005.pptx]

## Slide 1
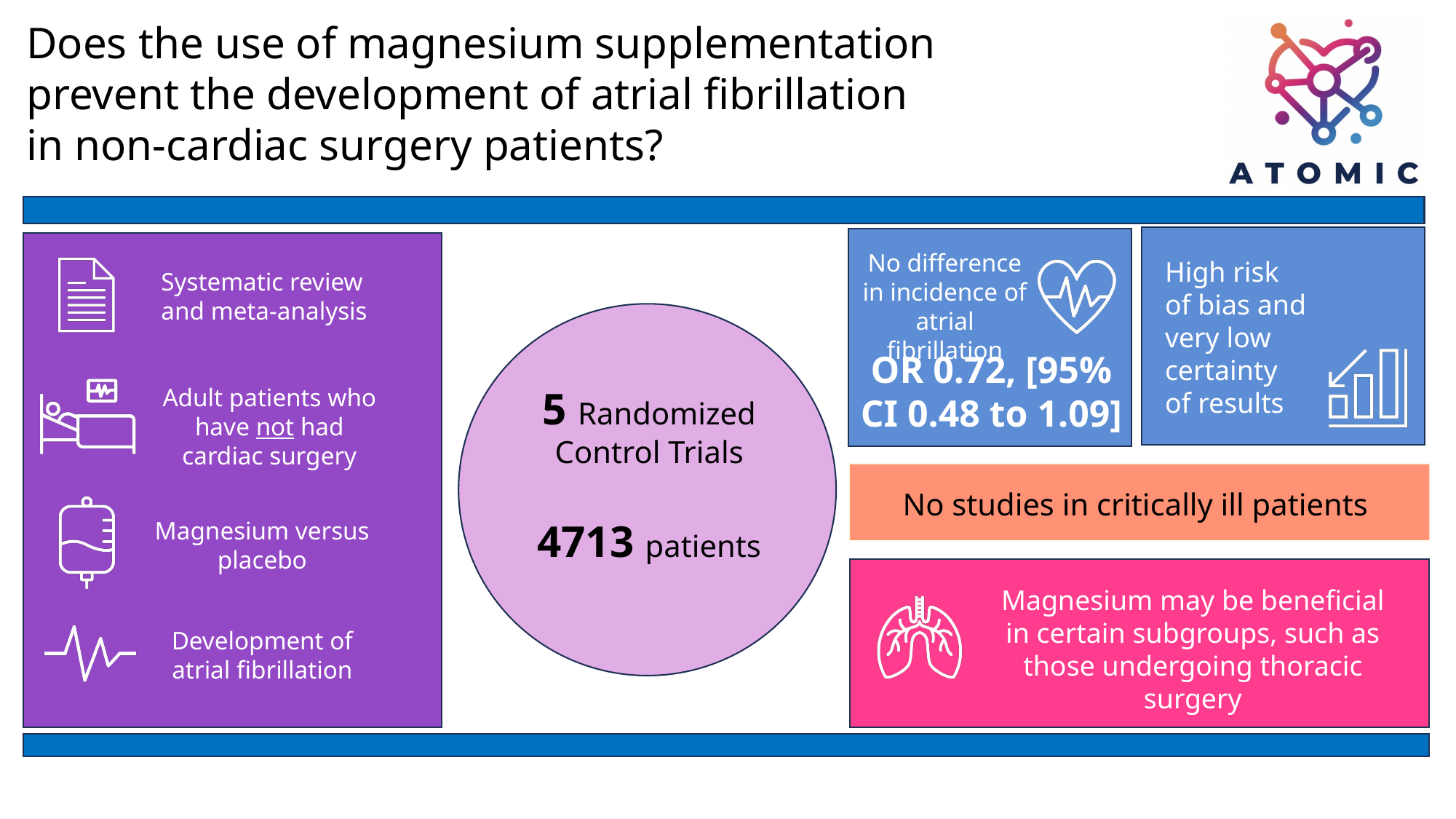

Does the use of magnesium supplementation
prevent the development of atrial fibrillation
in non-cardiac surgery patients?
No difference in incidence of atrial fibrillation
High risk of bias and very low certainty of results
Systematic review and meta-analysis
OR 0.72, [95% CI 0.48 to 1.09]
Adult patients who have not had cardiac surgery
5 Randomized Control Trials
No studies in critically ill patients
4713 patients
Magnesium versus placebo
Magnesium may be beneficial in certain subgroups, such as those undergoing thoracic surgery
Development of atrial fibrillation
